# Ready for a long fight against the COVID-19 outbreak: an innovative model of tiered primary health care in Taiwan

**DOI:** 10.3399/bjgpopen20X101068

**Published:** 2020-04-08

**Authors:** Brian Bih-Jeng Chang, Tai-Yuan Chiu

**Affiliations:** 1 Director, Brian's Family Doctor Clinic, New Taipei City, Taiwan; 2 Deputy Secretary General, Taiwan Medical Association, Taipei, Taiwan; 3 Professor of Family Medicine, College of Medicine, National Taiwan University, Taipei, Taiwan; 4 President, Taiwan Medical Association, Taipei, Taiwan

**Keywords:** COVID-19, primary healthcare, Taiwan, coronavirus

Between January 15 and March 31 2020, Taiwan reported 31 800 subjects tested, 322 confirmed COVID-19 cases (including 276 imported and 46 indigenous), and five deaths. Taiwan has been able to control the epidemic more effectively than many other countries in the Asia-Pacific region through a combination of measures, including border control; testing and quarantine of individuals with history of contacts; at-home self-isolation; and real-time linking of immigration records with healthcare information. Society maintains trust in governmental agencies thanks to daily press conferences, with full disclosure of key metrics and clear guidelines.^1^ An average of 675.76 tests per million individuals were performed. Our containment strategy ensured the numbers of new cases per day remain in single digits, delaying peak time and protecting the medical system from being overwhelmed. While first responses by primary health care is slowing down the outbreak, the Taiwan Medical Association (TMA) has devised a long-term strategy to handle the inevitable scenario of community transmission. Our plan relies on a tiered primary healthcare network of community healthcare groups prepared clinics (CHGPC)^2^ and community screening stations (CSS) to treat patients with mild symptoms at community clinics so hospitals and medical centres can focus on serious cases. Close coordination of hospitals and community care providers is key to guard the medical system against possible collapse due to sudden outbreaks of unknown pathogens.

More than 90% of the clinics in Taiwan participate in the National Health Insurance and accept walk-in patients. This provides a venue for rapid responses including education, diagnosis, isolation, and referral to de-escalate the virus outbreak. The importance of an effective community clinic is clear from the observation that between February 12 and March 13, 60% of the citizens returning from high-risk areas volunteered to visit community clinics (694 633 visits) (National Health Insurance Administration, Ministry of Health and Welfare Taiwan, *The Statistical Analysis of Outpatients' Clinical Data from NHI MediCloud system,* unpublished report, 2020).

**Figure 1.**
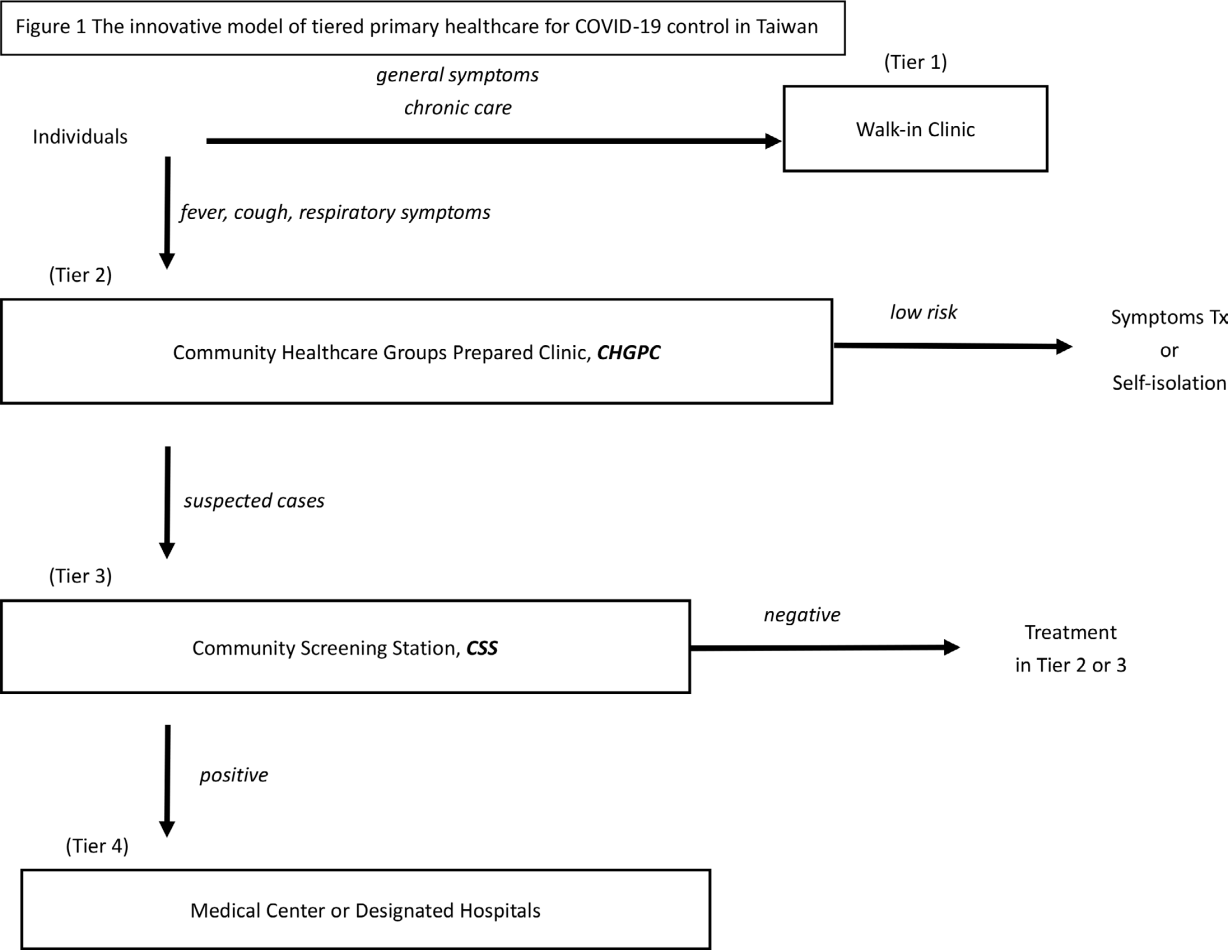
The innovative model of tiered primary healthcare for COVID-19 control in Taiwan.CHGPC = community healthcare groups prepared clinics. CSS = community screening stations. Tx = Treatment.

The Taiwanese primary healthcare model consists of four tiers (Figure 1):

- **Tier 1: Walk-in clinics** are equipped with standard protection equipment and provide general diagnostic and treatment services including chronic diseases, long-term care, preventive care, mental health care, wound care, and management of unknown symptoms.
- **Tier 2: Community Healthcare Groups Prepared Clinics** (CHGPC) accept patients with fever, cough, upper respiratory symptoms, or possible COVID-19 cases. CHGPCs provide the same services as walk-in clinics and can also monitor isolated cases with video conference calls. CHGPCs have reinforced protection. Participation is entirely voluntary. The government provides protective equipment and subsidies in order to recruit at least 20% of the clinics to participate in the programme.
- **Tier 3 Community Screening Stations** (CSS) consist of community health centres, regional hospitals, and other volunteering clinics that satisfy the programme requirements. CSSs are equipped with x-ray devices and can test and quarantine possible cases referred from CHGPCs. Confirmed cases could be treated locally (mild cases) or referred to the next tier. So far, 167 CSSs are active or being planned to cover all regions.
- **Tier 4: Medical Centres** are hubs of the network and treat referred confirmed cases with serious symptoms. They also test suspected cases and deliver routine services that are not available to regional hospitals and clinics.

Through this model, we are able to tackle the epidemic within the existing capacity of our medical system, without sacrificing important daily acute and chronic care functions in the community.

Community Healthcare Groups were introduced after the 2003 SARS (severe acute respiratory syndrome) epidemic, and they form the backbone of the primary care network in Taiwan as the first responders to public health emergencies.^2^ With lessons from SARS and hard-earned know-how in fighting against the viral epidemic, the municipal primary care network of 284 CHGPCs and 12 health centers under the Taipei Health Bureau handled the 2009 H1N1 pandemic effectively and avoided community transmission in the city.^3^ The episode gave our primary healthcare professionals valuable hands-on experience, which was instrumental in their timely and effective responses at the beginning of the COVID-19 outbreak. As COVID-19 inevitably becomes a global pandemic, a robust tiered primary care network capable of agile reactions is key to respond to the outbreak while maintaining other important medical care functions. The TMA strategy is a good example of a collaboration between governmental and private healthcare networks. We hope our experience will be useful for other countries to implement more effective measures to fight against the spread of COVID-19.
